# Clonal Evolution of B-Cell Acute Lymphoblastic Leukemia with del(9)(p13p21) into Mixed Phenotype Acute Leukemia Presenting as an Isolated Testicular Relapse

**DOI:** 10.3390/reports2030018

**Published:** 2019-07-15

**Authors:** Lane H. Miller, Sunita I. Park, Debra Saxe, Glen Lew, Sunil S. Raikar

**Affiliations:** 1 Department of Pediatrics, Aflac Cancer and Blood Disorders Center, Children’s Healthcare of Atlanta and Emory University, Atlanta, GA 30322, USA; 2 Cancer and Blood Disorders Center, Children’s Minnesota, Minneapolis, MN 55404, USA; 3 Department of Pathology, Children’s Healthcare of Atlanta, Atlanta, GA 30322, USA; 4 Department of Pathology and Laboratory Medicine, Emory University, Atlanta, GA 30322, USA

**Keywords:** B-ALL, MPAL, clonal evolution, del(9)(p13p21)

## Abstract

Lineage switch in acute leukemias is a well-reported occurrence; however, most of these cases involve a switch from either lymphoid to myeloid or myeloid to lymphoid lineage. Here, we report a case of a 14-year-old male with B-cell acute lymphoblastic leukemia (B-ALL) who initially responded well to standard chemotherapy but then later developed mixed phenotype acute leukemia (MPAL) at relapse, likely reflecting a clonal evolution of the original leukemia with a partial phenotypic shift. The patient had a del(9)(p13p21) in his leukemia blasts at diagnosis, and the deletion persisted at relapse along with multiple additional cytogenetic aberrations. Interestingly, the patient presented with an isolated testicular lesion at relapse, which on further analysis revealed both a lymphoid and myeloid component. Unfortunately, the patient did not respond well to treatment at relapse and eventually succumbed to his disease. To our knowledge, an isolated extramedullary MPAL at relapse in a patient with previously diagnosed B-ALL has not been reported in the literature before.

## 1. Introduction

Mixed phenotype acute leukemia (MPAL) is a rare but well-described form of childhood leukemia, with the leukemic blasts expressing features of both lymphoid and myeloid lineage [1–5]. These cases typically have a de novo presentation, with the initiating cell believed to be an early common lineage precursor cell. Lineage switch in acute leukemias is a well-reported phenomenon, believed to be related to the plasticity of the initiating leukemia stem cell [6]. However, most of these cases involve a switch from either lymphoid to myeloid or myeloid to lymphoid lineage [6]. B-cell acute lymphoblastic leukemia (B-ALL) transforming into MPAL at relapse, however, is an extremely rare event and likely reflects a clonal evolution of the original leukemia with a partial phenotypic shift. Moreover, isolated extramedullary MPAL at relapse in a previously diagnosed B-ALL case is to our knowledge entirely undocumented in the literature.

Chromosomal 9p deletions are relatively frequent in childhood B-ALL, occurring in roughly 10% of cases and portending an adverse outcome when compared to cases without this anomaly [7]. The 9p21 region has been heavily investigated and has been shown to harbor critical tumor suppressor genes such as *CDKN2A/B* [8–10]. Herein, we present the case of an adolescent male with immunophenotypically characteristic B-ALL exhibiting a del(9)(p13p21) on conventional cytogenetic analysis, who initially presented with marrow disease alone and attained remission with standard B-ALL therapy. He later developed an isolated MPAL testicular relapse followed by bone marrow involvement and the subsequent development of myeloid sarcomas and leukemia cutis, all while persistently harboring the del(9)(p13p21) with additional cytogenetic aberrations.

## 2. Case Presentation Section

**Figure 1.**
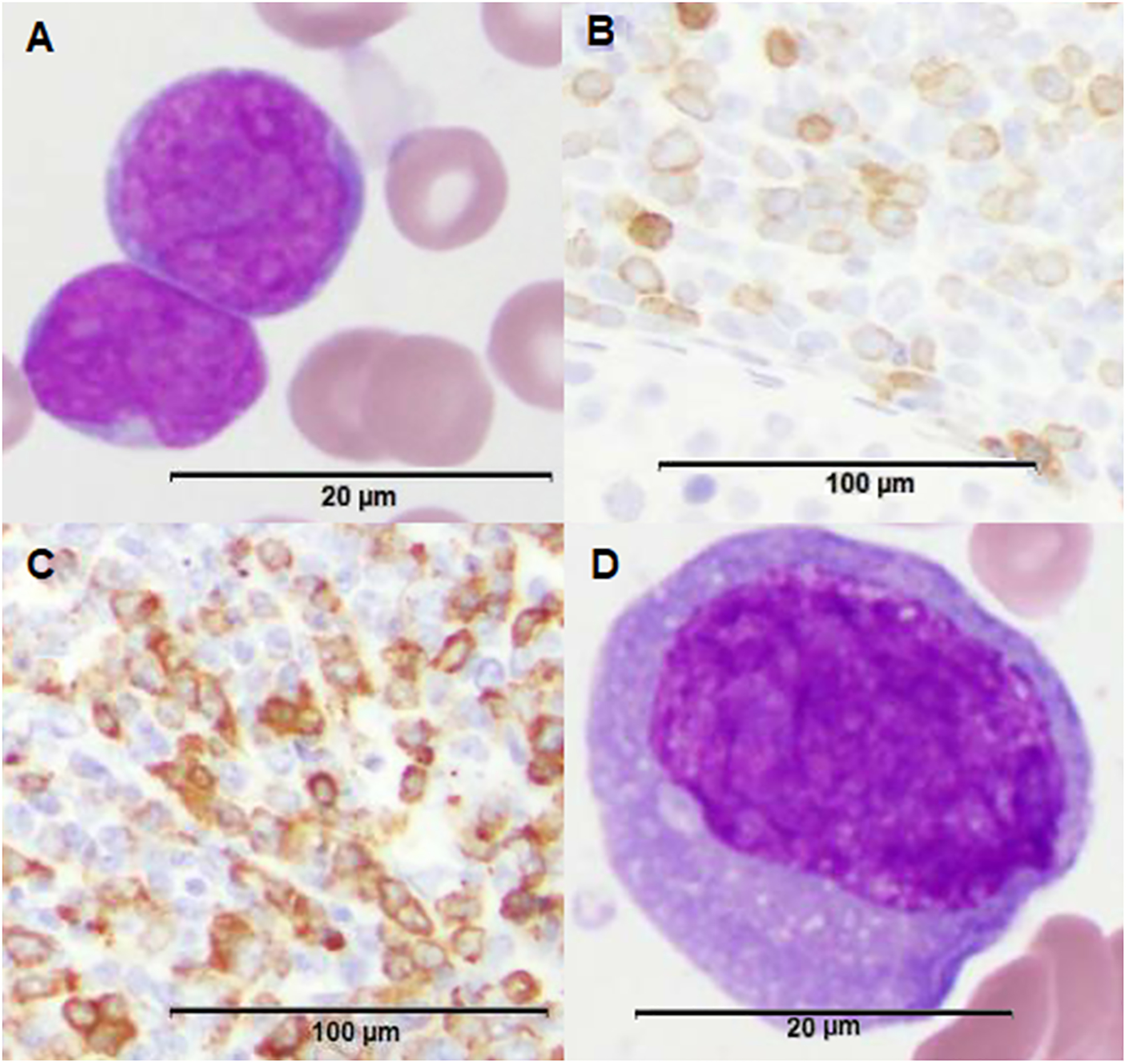
Morphologic features of patient’s leukemia at various time points and across different sites of disease. (**A**) H&E stain of bone marrow biopsy demonstrating B-cell acute lymphoblastic leukemia (B-ALL) blasts (100x magnification). (**B**) CD79a stain of testicular biopsy demonstrating B-lymphoid component within the isolated testicular mixed phenotype acute leukemia (MPAL) relapse (20× magnification). (**C**) Myeloperoxidase (MPO) stain of testicular biopsy demonstrating a larger myeloid component within the isolated testicular MPAL relapse (20× magnification). (**D**) H&E stain of bone marrow biopsy upon predominantly myeloid marrow relapse demonstrating a myeloblast (100× magnification).

**Figure 2.**
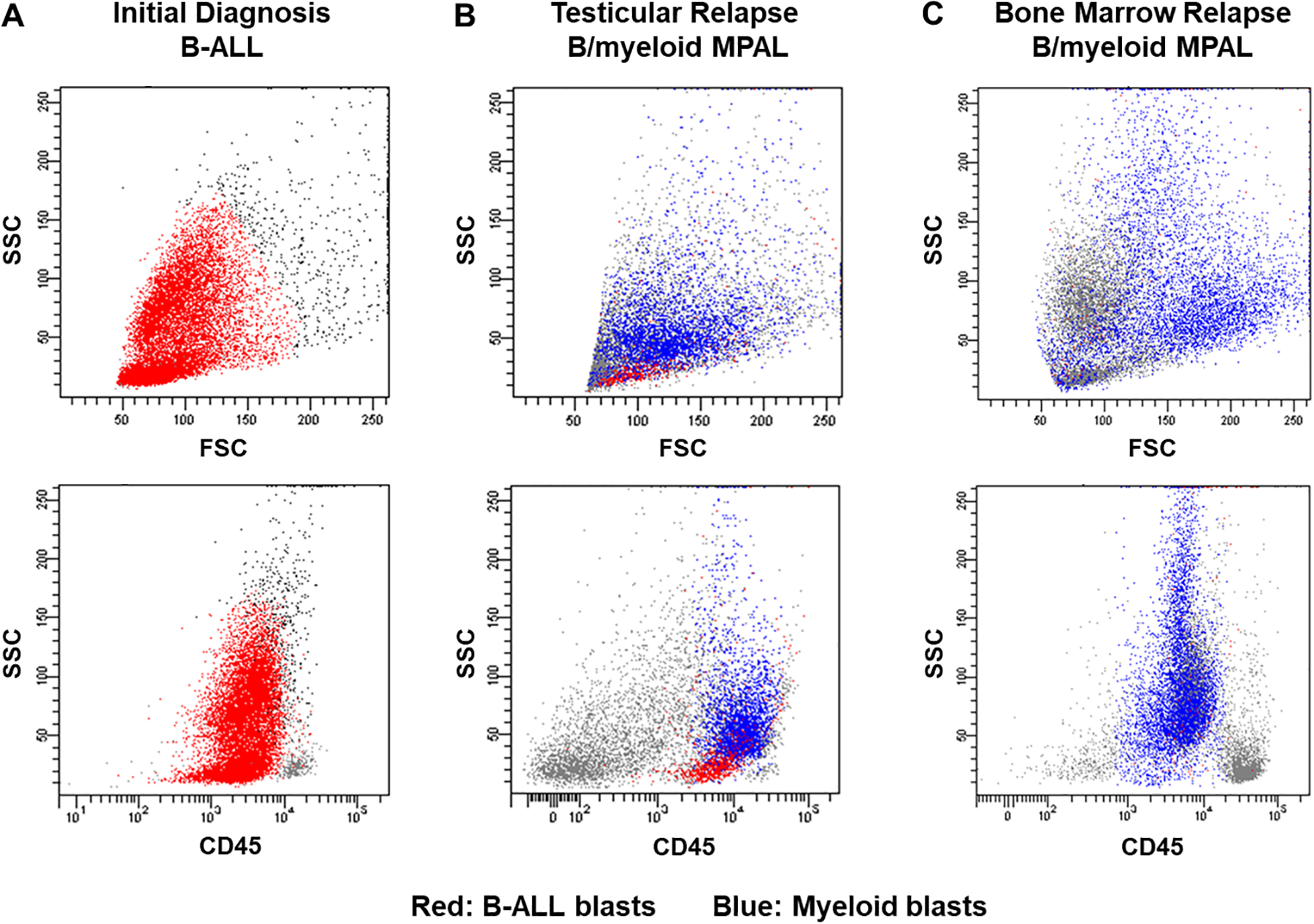
Flow cytometry dot plot representation of the patient’s leukemia at various time points and across different sites of disease. The top three panels demonstrate the patient’s leukemia blasts along the side scatter (SSC) vs. forward scatter (FSC) axis at (**A**) initial diagnosis, (**B**) testicular relapse, and (**C**) first bone marrow relapse, whereas the bottom three panels depict the blasts along the SSC vs. CD45 axis at the same time points. B-ALL blasts are represented in red, while the myeloid lineage blasts are depicted in blue.

**Figure 3.**
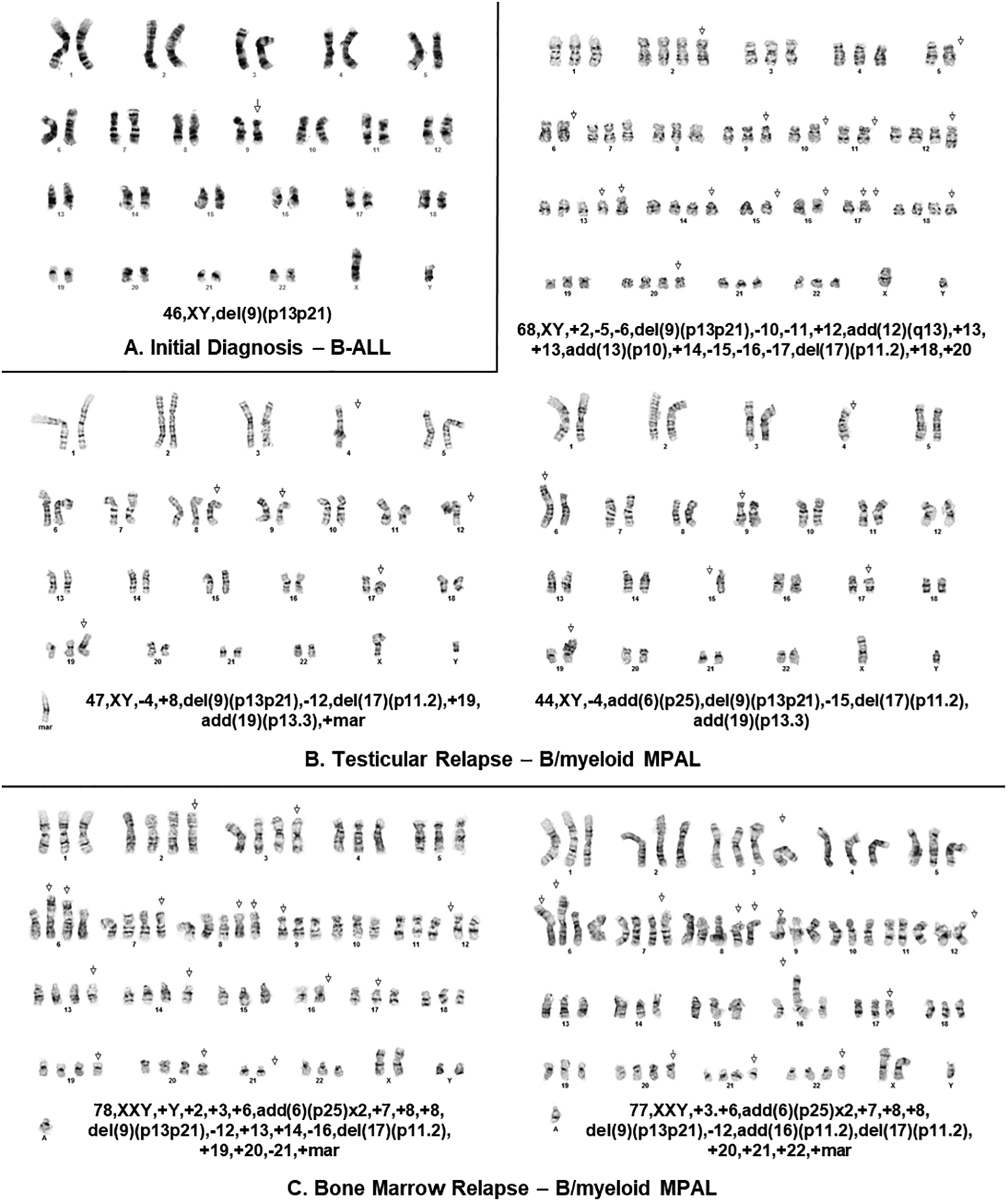
Karyotype of patient’s leukemia at various time points and across different sites of disease. (**A**) Cytogenetic analysis of B-ALL blasts at diagnosis obtained from a bone marrow aspirate and demonstrating 46,XY,del(9)(p13p21) in all cells analyzed. (**B**) Cytogenetic analysis of biopsy sample obtained from testicular relapse also demonstrating the del(9)(p13p21) in most cells analyzed among additional anomalies. (**C**) Cytogenetic analysis of MPAL blasts at marrow relapse obtained from a bone marrow aspirate and demonstrating hyperdiploidy, del(9)(p13p21) and additional aberrations.

A 14-year-old male presented with a two-month history of fatigue, malaise, and weight loss. His complete blood count revealed a white blood cell (WBC) count of 44,000 cells/μL and 94% peripheral blasts (Figures 1a and 2a). The diagnosis of B-ALL was confirmed by peripheral blood flow cytometry. A bone marrow aspirate yielded 96% B-lymphoblasts with a fairly typical B-ALL immunophenotype (Table 1), although interestingly, the partial CD10 and CD20 expression suggested a partly early pre-B-ALL or pro-B-ALL phenotype. Antigen expression for all markers was reported in accord with consensus recommendations from clinical flow cytometry experts in the field [11,12]. Cytogenetics obtained prior to chemotherapy initiation demonstrated 46,XY,del(9)(p13p21) in all cells analyzed (Figure 3a). His cerebrospinal fluid (CSF) was negative for blasts. Physical examination at diagnosis demonstrated bilaterally descended testes with no masses. Given his presenting age, he was classified as National Cancer Institute (NCI) high risk B-ALL and was treated with four-drug induction chemotherapy. He attained complete remission by the end of induction as defined by a bone marrow minimal residual disease (MRD) level of less than 0.01%. Post-induction chemotherapy continued per conventional Children’s Oncology Group (COG) protocols.

Approximately two years from diagnosis, the patient developed left sided scrotal pain determined to be a mass on ultrasound. A testicular biopsy revealed the presence of two distinct abnormal cell populations, including B-lymphoblasts and monocytic lineage cells (Figures 1b and 2b, Table 1) consistent with a bilineal form of MPAL. Cytogenetic findings again demonstrated the del(9)(p13p21) in most cells analyzed, along with additional abnormalities, suggesting a clonal evolution of the prior B-ALL (Figure 3b). The relapsed B-lymphoblasts were negative for both CD10 and CD20 expression, suggesting the evolution of a clone with a more immature phenotype. Microarray analysis of the testicular tissue confirmed the persistence of del(9)(p13p21). Bone marrow and CSF evaluations at relapse showed no evidence of disease. He was treated with a lymphoid-based salvage chemotherapy regimen designed for isolated extramedullary relapse, which included high-dose methotrexate followed by a modified re-induction with mitoxantrone, vincristine, and dexamethasone. Consolidation therapy included high-dose cytarabine in conjunction with testicular radiotherapy. Of note, due to allergies to both pegylated E. coli and Erwinia asparaginase, this agent was omitted from his chemotherapy regimen.

His ensuing course was complicated by the development of a left gluteal mass four months after his testicular relapse, found to be a granulocytic sarcoma with diffuse positivity for CD43, CD56, and CD33 and focal positivity for CD68 and MPO on immunohistochemical staining (Figure 1c). Concurrent heart block was presumed to be secondary to cardiac chloromas. Repeat bone marrow evaluation at that time demonstrated MPAL with a larger population (38% of the sample) consisting of acute myeloid leukemia (AML) with monocytic differentiation and a much smaller population (0.4% of the sample) consisting of B-ALL (Figures 1d and 2c, Table 1). Cytogenetic analysis at that time continued to be remarkably different than at diagnosis and initial relapse, with 10 abnormal metaphases demonstrating hyperdiploidy (76 to 78 chromosomes per cell), del(9)(p13p21), and numerous additional chromosomal aberrations (Figure 3c). Testing for *FLT3* internal tandem duplication was negative. Subsequent management with one cycle of mitoxantrone and high-dose cytarabine yielded a marrow remission, however, he concomitantly developed violaceous nodules on the legs, arms, and abdomen as well as a pericardial effusion, respectively shown to be leukemia cutis on biopsy (myeloid lineage, CD33 positive on immunohistochemistry) and monocytic AML infiltration on pericardial fluid immunophenotyping. Palliative radiation was provided to the chest. Repeat bone marrow aspirate less than two weeks following the prior marrow evaluation showed the presence of 23% involvement by B-ALL, with blasts similar to those seen previously. After lengthy prognostic discussions, he was discharged to home hospice care and ultimately succumbed to his disease.

## 3. Discussion

The case herein describes a unique presentation of clonal evolution of B-ALL to MPAL, MPAL presenting on relapse as an isolated testicular mass, and a del(9)(p13p21) serving in some capacity as a driver mutation at diagnosis and relapse.

To our knowledge, isolated testicular MPAL at relapse is an entirely undescribed entity. In fact, while isolated testicular relapse is relatively common in childhood ALL (B and T-cell), occurring in approximately 2% of cases and predominantly in the late range of relapse (>36 months from achieving complete remission) [13], isolated testicular AML relapse is extraordinarily rare in children [14,15]. The most recent comprehensive review of isolated testicular relapse in childhood AML noted 16 described cases, with initial French-American-British (FAB) subtypes including M0 (*n* = 2), M1 (*n* = 1), M2 (*n* = 2), M4 (*n* = 8), M5 (*n* = 1), and not otherwise specified, NOS (*n* = 2) [14].

B-ALL evolving into MPAL has only once been described in the literature before. Carulli et al. reported a case of a 21-year-old female with B-ALL associated with t(4;11)(q21;q23) who was treated with the Hyper-CVAD regimen, and during the course of therapy (after 6 of 8 planned cycles) had evolved into MPAL with additional chromosomal aberrations [16]. In regards to the incidence of lineage switch from B-ALL to frank AML at relapse, only sparse reports exist, most of which are associated with *KMT2A*-rearranged infant B-ALL [17–26]. In 2012, Dorantes-Acosta and Elayo noted 18 documented cases of lineage switch in the pediatric acute leukemia literature, including B-ALL to AML (*n* = 10), AML to B-ALL (*n* = 5), T-ALL to AML (*n* = 2), and B-ALL to T-ALL to AML (*n* = 1) [6]. In the case at hand, the del(9)(p13p21) persisted from the B-ALL at diagnosis to the testicular MPAL at relapse and ultimately in the predominantly myeloid marrow relapse several months later, all of which indicated clonal evolution within both the lymphoid and myeloid lineages. Interestingly, unlike the initial presenting leukemia with partial CD10 and CD20 expression, the relapsed clone was both CD10 and CD20 negative, suggesting it had evolved from a more immature B-lymphoblast, likely one with more plasticity.

Cytogenetic 9p abnormalities are independent prognostic indicators of inferior outcomes in childhood B-ALL [7]. The 9p21.3 region contains well-known tumor suppressor genes including the *cyclin-dependent kinase inhibitor 2A/B* (*CDKN2A/B*) gene [8,9]. *CDKN2A/B* encodes three proteins involved in tumor suppressor pathways - p14^ARF^, p15^INK4B^, and p16^INK4A^. p15^INK4B^ binds to MDM2, thus stabilizing the tumor suppressor protein p53, whereas p14^ARF^ and p16^INK4A^ selectively inhibit cyclin-D dependent kinases CDK4 and CDK6, thus preventing phosphorylation of RB1 [9,10]. Thus, deletions in the *CDKN2A/B* gene disrupt both the p53 and RB1 tumor suppressor pathways. The prognostic value of *CDKN2A/B* deletions in ALL has been extensively studied by multiple groups, with most studies reporting it to be associated with inferior outcomes [9,10]. While not as prevalent as in ALL, the *CDKN2A/B* deletion has also been reported in AML [27,28]. Our current method of cytogenetic analysis does not sort MPAL cases by lineage type, thus we cannot say with certainty whether both the lymphoid and myeloid populations contained the del(9)(p13p21). However, in this case, with the deletion being present at B-ALL diagnosis and in 62.5% (10/16) of metaphases analyzed in a predominantly myeloid marrow relapse (38% monocytic and 0.4% B-ALL), it is highly suggestive of a del(9)(p13p21) within both populations. While the exact driving mutation underlying this patient’s leukemia cannot be determined, it is reasonable to assume that with the persistence of this cytogenetic aberration, this tumor suppressor gene is implicated.

Finally, as seen in this case, MPAL can be particularly difficult to treat and may require novel therapies in order to improve survival [5]. Current leukemia regimens are primarily lineage specific and tailored toward either lymphoid or myeloid disease. Although recent literature suggests that MPAL patients respond better to ALL directed therapy [29–31], the risk in doing so is that a potentially untreated myeloid clone may be unwittingly allowed to expand, especially in bilineal cases such as this involving separate lymphoid and myeloid blast populations [31,32]. Additionally, MPAL presenting at relapse is indicative of clonal evolution secondary to lineage plasticity and signifies aggressive and potentially chemo-resistant bilineal clones. In the era of precision medicine and common application of DNA microarray and genomic analysis of leukemic blasts at diagnosis and relapse, targeted therapeutic options are becoming more frequently available [33]. In the case of true 9p21.3 deletions, small molecule inhibitors of CDK4 and CDK6 have been explored and should certainly be considered as potential adjuncts to standard therapy [34].

## Figures and Tables

**Table 1.** Immunophenotypic characteristics of the patient’s initial diagnostic bone marrow, testicular relapse, and bone marrow relapse.

Flow cytometry marker	B-ALL bone marrow at diagnosis	Testicular relapse (2 years from diagnosis)		Bone marrow relapse (4 months from testicular relapse)	
		B-ALL component	Myeloid component	B-ALL component (0.4% cells)	Myeloid component (38% cells)
CD2	−	−	+	−	−
CD4	−	−	+	−	+
CD10	+	−	−	−	−
CD11b	−	−	+	−	+
CD13	+	+	+	−	+
CD14	−	−	+	−	−
CD15	−	−	+	−	−
CD19	+	+	−	+	−
CD20	+	−	−	−	−
CD22	+	+	−	+	−
CD33	−	−	+	−	+
CD34	+	+	−	+	−
CD36	−	−	+	−	−
CD38	+	+	+	+	+
CD56	−	−	−	−	+
CD58	+	+	+	+	+
CD64	−	−	+	−	+
MPO	−	−	+	−	+
